# Evaluation of Genome Sequences of the Bacteriophages JeTaime and Luna22

**DOI:** 10.1128/MRA.00746-21

**Published:** 2021-10-07

**Authors:** Maximiliano F. Flota, Caitlyn R. Moss, Mitchell F. Balish, Nikki Chen, Kristen C. Cherry, Kathleen A. Cornely, Hayley M. D’Alessandro, Mouna S. DiBenedetto, Joseph A. DeGiorgis, Steven Grant Dixon, Emily G. Dombrowski, Megan K. Edwards, Jonathan C. Eskew-Martin, Isabel E. P. Finnegan, Abanob G. Hanna, Sarah E. Hunter, Sabrina L. Johnson, Virginia A. L. Kenan, Chanelle Kendrick, Lucas C. Licaj, Victoria C. Maldonado, Megan G. Mazzei, Gracen E. Mitrick, Bradford L. J. Nelson, Jui S. Patel, Alexander I. Parry, Kacey M. Smekrud, Kirsten K. Snyder, Jonathan A. Stewart, Fredrick K. Swiger, Madison K. Thomas, Josiah C. Waters, Christine A. Byrum

**Affiliations:** a Department of Biology, College of Charleston, Charleston, South Carolina, USA; b Department of Microbiology, Miami University, Oxford, Ohio, USA; c Department of Chemistry and Biochemistry, Providence College, Providence, Rhode Island, USA; Portland State University

## Abstract

The mycobacteriophages JeTaime (E cluster) and Luna22 (Q cluster) were isolated from soil in Providence, Rhode Island, and Charleston, South Carolina, respectively, using a Mycobacterium smegmatis mc^2^ 155 host. The genome of JeTaime is 75,099 bp (142 predicted genes), and that of Luna22 is 53,730 bp (87 predicted genes). Both phages exhibit *Siphoviridae* morphology.

## ANNOUNCEMENT

**FIG 1:**
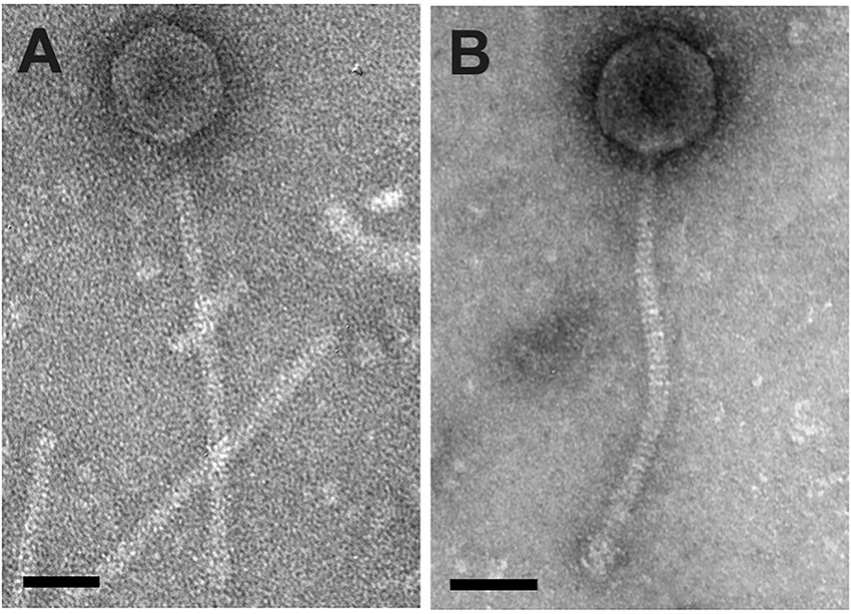
JeTaime (A) and Luna22 (B) morphology, examined using transmission electron microscopy. High-titer lysates placed on Formvar-coated grids were negatively stained with 1% uranyl acetate (2). JeTaime was imaged using a JEOL 20× transmission electron microscope (100 kV), and Luna22 was imaged using a JEOL 1010 transmission electron microscope (80 kV). Bars, 50 nm.

Bacteriophage genome characterization provides key insights into viral evolution and genetic diversity for both basic and applied research. The mycobacteriophages JeTaime and Luna22 were investigated in partnership with the Science Education Alliance-Phage Hunters Advancing Genomics and Evolutionary Science (SEA-PHAGES) program through the Howard Hughes Medical Institute (HHMI) (1). The phage JeTaime was found in peaty surface soil from Providence, Rhode Island, and Luna22 was found in loamy surface soil from Charleston, South Carolina (Table 1). Both phages were isolated using enrichment at 37°C, followed by purification and amplification in Mycobacterium smegmatis mc^2^ 155, as described in the SEA-PHAGES Phage Discovery Guide (2). Cultured viruses formed clear, circular plaques, and electron microscopy revealed *Siphoviridae* morphotypes with icosahedral capsids and long flexible tails (Fig. 1).

**TABLE 1:** Characteristics of the JeTaime and Luna22 phages

Phage name	GenBank accession no. (SRA no.)	Location	Genome size (bp)	Coverage (×)	% GC	No. of tRNAs (no. of tmRNAs)	Avg plaque diameter (mm)	Capsid size (nm)	Tail length (nm)
JeTaime	MZ322024.1 (SRX11158995)	Providence, RI (41.840000N, 71.40000W)	75,099	1,389	62.9	2 (0)	1.5–2.9, x̄ = 1.9 (*n* = 10)	75.0–78.0, x̄ = 75.4 (*n* = 4)	275–312.5, x̄ = 296 (*n* = 3)
Luna22	MW924639 (SRX11158996)	Charleston, SC (32.784094N, 79.935882W)	53,730	2,352	65.7	0 (0)	2.0–3.0, x̄ = 2.5 (*n* = 7)	62.5–75.0, x̄ = 70 (*n* = 5)	231.3–262.5, x̄ = 244 (*n* = 5)

The Promega Wizard DNA cleanup system was used to extract DNA from phage lysates, and sequencing libraries were prepared with a NEBNext Ultra II DNA library prep kit (v3). Libraries for each phage were independently generated, and genomes were sequenced separately. Sequencing was performed at the Pittsburgh Bacteriophage Institute on an Illumina MiSeq system (MiSeq reagent kit v3) (3), and 150-bp single-end reads were collected (73,757 reads for JeTaime and 878,986 reads for Luna22). Raw reads were then assembled *de novo* into a single contig using Newbler v2.9 (4) and verified with Consed v29.0 (5), as described by Russell (3). Using PAUSE (https://cpt.tamu.edu/computer-resources/pause), genome termini with 3′ cohesive overhangs were identified, as follows: JeTaime, 5′-CGCTTGTCA-3′; Luna22, 5′-TAAGCCGCGCGGTA-3′. Table 1 summarizes additional findings.

Each genome was annotated on PECAAN (6) using GLIMMER v3.02 (7), GeneMark v2.5 (8), and Starterator v1.1 (9). To refine start sites, to determine clusters, and to compare related genomes, Phamerator (10, 11) was utilized. Functional assignments were made with BLASTp v2.9 (12), HHpred (13), TMHMM2 (http://www.cbs.dtu.dk/services/TMHMM), TOPCONS v2 (14), and the NCBI Conserved Domain Database (CDD) (15). tRNAs and transfer-messenger RNAs were identified with ARAGORN v1.2.38 (16) and tRNAscan-SE v3.0 (17), and completed files were transferred to DNA Master v5.22.2 (https://phagesdb.org/DNAMaster). All programs used default settings.

JeTaime is an E-cluster phage with 142 predicted protein-coding genes (54 assigned functions), while Luna22 is a Q-cluster phage with 87 predicted protein-coding genes (42 assigned functions) (11). Interestingly, sequence upstream of the single-stranded DNA (ssDNA)-binding protein (gp99) is absent in the JeTaime and NelitzaMV (GenBank accession number KT222941) genomes. The other 103 published E-cluster members typically contain endonuclease VII and/or DNA methylase in that genome region. Also, the JeTaime gp89 (unidentified function) is rare, occurring in only one other E-cluster bacteriophage, 244 (GenBank accession number DQ398041). Whole-genome BLASTn alignment (12) revealed that the JeTaime genome is most similar to genomes of the E-cluster phages Cjw1 (GenBank accession number NC_004681.1) (99.24% identity and 96% coverage), Phrux (GenBank accession number NC_021309.1) (99.20% identity and 95% coverage), and ShereKhan (GenBank accession number MH513983.1) (99.10% identity and 95% coverage).

Interestingly, Luna22 and other Q-cluster genomes exhibit very high levels of conservation. Whole-genome BLASTn alignment (12) revealed that 13/15 published Q-cluster genomes share >99.80% identity with the Luna22 genome (100% coverage). Q-cluster phages from as far away as Washington state (Ein37) (GenBank accession number MT114159) and New Zealand (Daegal) (GenBank accession number MK494095) retain >98% nucleotide sequence identity to the Luna22 genome. Also, the genome of Giles (GenBank accession number EU203571) is nearly identical to that of Luna22 (99.96% identity and 100% coverage) (18–21); the only difference is that bases 33464 to 33479 in Giles are missing in Luna22. Loss of this sequence results in a frameshift mutation, causing gp42 (DNA-binding protein) to be truncated in Luna22 after amino acid 215.

### Data availability.

Individual GenBank and SRA numbers are listed in Table 1.
